# Fatal abdominal hemorrhage following surgery to remove a retroperitoneal MPNST associated with *NF1*: A case report

**DOI:** 10.1097/MD.0000000000040745

**Published:** 2024-11-29

**Authors:** Yu-Yang Pei, Tian-Tong Yang, Hai-dong Zhang, Tian-Shui Yu

**Affiliations:** a Key Laboratory of Evidence Science, Ministry of Education, China University of Political Science and Law, Beijing, China; b Center of Cooperative Innovation for Judicial Civilization, Beijing, China.

**Keywords:** abdominal hemorrhage, heterogeneous differentiation, malignant peripheral nerve sheath tumors, neurofibromatosis type I

## Abstract

**Rationale:**

Individuals diagnosed with neurofibromatosis type I (NF1) commonly present with neurofibromas, and a subset may progress to develop malignant peripheral nerve sheath tumors (MPNST) during their lifetime. Diagnosing and treating MPNST, typically linked to NF1, poses challenges for clinicians due to its histopathological complexity.

**Patient concerns:**

A 25-year-old male presented with postprandial discomfort and vomiting, subsequently developing left mid-abdominal pain.

**Diagnoses:**

The patient was admitted to the hospital, where a low-density retroperitoneal mass was detected via computed tomography (CT). Histopathological examination revealed spindle-shaped tumor cells characterized by abundant cytoplasm and highly pigmented nuclei, demonstrating pathological nuclear division. The tumor cells exhibited partial cytoplasmic positive for S-100 and focal cytoplasmic positive for cytokeratin (CK) and desmin, as determined by immunohistochemical staining. Genetic analysis of blood and extracted tissues identified an *NF1* missense mutation. Prior research corroborated the pathological diagnosis of MPNST exhibiting both epithelial and myogenic differentiation.

**Interventions:**

A retroperitoneal mass excision was conducted, revealing a mass located in the retroperitoneal omental sac.

**Outcomes:**

Approximately 5 hours after surgery, the patient’s blood pressure exhibited a gradual decline. An emergency laparotomy was conducted. Approximately 3000 mL of blood was identified in the upper abdominal cavity. The patient’s blood pressure consistently declined and ultimately resulted in death after 2 days.

**Lessons:**

It is crucial to assess the potential for heterogeneous differentiation in MPNST during pathological diagnosis. In the treatment of MPNST with heterogeneous differentiation, particularly in cases with significant tumor bulk, surgeons must anticipate potential hemorrhagic complications and adopt a cautious approach to surgical intervention.

## 1. Introduction

Neurofibromatosis type 1 (NF1) is a genetic disorder transmitted in a dominant fashion due to mutations in the *NF1* gene, responsible for the production of the tumor suppressor neurofibromin. The *NF1* gene exhibits 1 of the highest rates of spontaneous mutation in the human genome, attributed to its considerable size of over 300 kilobases and 60 exons, which encompass multiple alternatively spliced exons. About 50% of *NF1* mutations arise de novo, while the other mutations are inherited. The prevalence of NF1 is 1 in every 2500 live births, categorizing it as 1 of the most prevalent single-gene inheritance disorders.^[1,2]^ Neurofibromin functions as a RAS GTPase-activating protein (RAS-GAP), regulating all RAS proteins.^[3]^ It is found in multiple cell types, especially those originating from the neural crest, such as neurons, cells of the nervous system, and early melanocytes. Consequently, almost all individuals with NF1 exhibit abnormalities involving the skin, central nervous system, and peripheral nervous system.

Malignant peripheral nerve sheath tumors (MPNST) represent 2% of soft-tissue malignant tumors and are found in 8% to 13% of individuals with NF1.^[4]^ In some cases, MPNST may arise from disseminated tumors or as a result of radiotherapy given for other types of tumors.^[5]^ Transitioning from neurofibroma to MPNST in cases associated with NF1 presents challenges due to the intralesional heterogeneity of these tumors, complicating radiological imaging and biopsy efforts. MPNSTs are linked to unfavorable survival rates and represent the primary cause of mortality among patients with NF1.^[6]^ Delayed diagnosis may lead to poor outcomes; however, ineffective treatment is the primary cause. Total surgical removal is the primary treatment for MPNST; however, it is often hindered by the considerable size of the tumors, their closeness to complex nerve networks, and a low rate of obtaining clear resection margins.^[7]^ Chemotherapy and ionizing radiation are applicable for unresectable and high-risk MPNSTs.

Severe perioperative complications resulting from the congenital fragility of the vascular wall in patients with NF1 have been reported, although such occurences are rare.^[8]^ To date, there have been no reports of hemorrhage following surgery for the removal of MPNST. This report presents a case of a patient who suffered fatal abdominal hemorrhage subsequent to surgery for the excision of a retroperitoneal MPNST. We have further clarified the pathogenic characteristics of MPNST, particularly improving our comprehension of MPNST with diverse differentiation.

## 2. Case report

**Figure 1.**
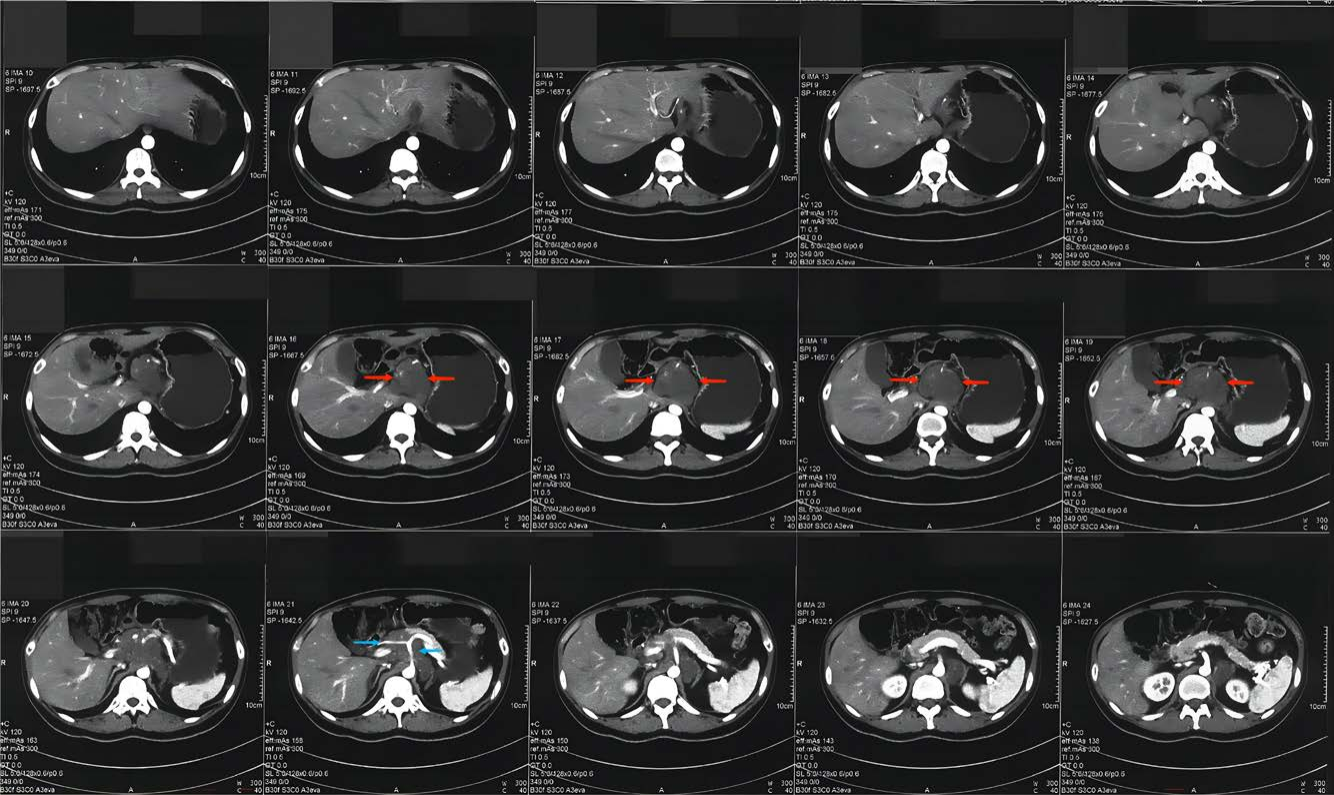
The abdominal CT scan demonstrates a low-density mass located in the retroperitoneum, as indicated by the red arrows. The mass encircles the celiac trunk and its branches, as illustrated by the blue arrows. CT = computed tomography.

A 25-year-old male of Han nationality, with a height of 160.0 cm and a weight of 55.0 kg, presented to our hospital with postprandial discomfort and vomiting. He is currently unemployed following his university graduation. Computed tomography (CT) identified an abdominal tumor (Fig. 1), while radiography revealed thoracic scoliosis. Surgery was conducted on the seventh day of hospitalization to excise the tumor situated in the retroperitoneal omental sac from 9:10 am to 2:15 pm The tumor encased the abdominal aorta, including the celiac trunk, and inferior vena cava. The tumor encased the common hepatic and left gastric arteries. Due to significant encirclement, complete separation of the tumor was not feasible. Consequently, the tumor situated in the retroperitoneum was excised in separate chunks. A segment of the surgically excised tumor was submitted for intraoperative cryopathological analysis, which identified spindle cell tumors. During the operation, the patient received a transfusion of 2 units of erythrocytes, 400 mL of plasma, and 400 mL of autologous blood. Approximately 5 hours after surgery, the patient exhibited a sudden and progressive decrease in blood pressure, ultimately measuring 50/40 mm Hg. The pulse oximetry reading decreased by 70%. An emergency laparotomy was conducted through the initial abdominal incision from 7:45 pm to 9:40 pm, revealing a significant volume of blood (approximately 3000 mL) in the upper abdominal cavity. The surgeon identified an enlarged pinhole in the vascular suture at the branch stump of the celiac trunk, accompanied by continuous rapid bleeding. The surgeon employed vascular sutures to promptly close the bleeding site and eliminate blood accumulation. The patient received a transfusion of 16 units of erythrocytes and 1400 mL of plasma. Upon return to the ICU, the patient’s blood pressure persisted in declining, leading to death after 2 days.

**Figure 2.**
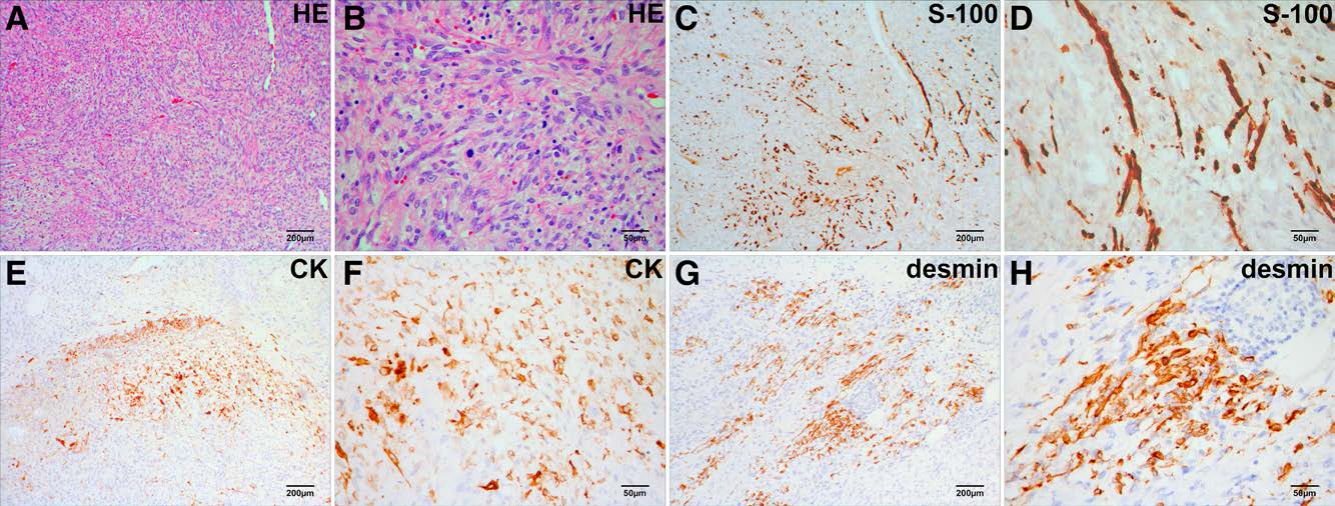
The MPNST is subjected to hematoxylin-eosin staining (A and B) and immunostaining (C–H). (A) The tumor cells exhibit dense packing, accompanied by uniform pink necrotic debris interspersed among them. (B) The tumor cells exhibit a spindle shape, possess abundant cytoplasm, and feature highly pigmented nuclei that demonstrate abnormal nuclear division. (C and D) The tumor cells demonstrated partial cytoplasmic positivity for S-100 protein. (E and F) The tumor cells exhibited localized cytoplasmic positivity for CK. (G and H) The tumor cells exhibited localized cytoplasmic positivity for desmin. CK = cytokeratin, MPNST = malignant peripheral nerve sheath tumors.

An autopsy was conducted 2 days postmortem to determine the specific characteristics of the tumor and the underlying cause of the hemorrhage.The body exhibited multiple café-au-lait spots on the skin, accompanied by several rounded cutaneous neurofibromas. A significant volume of bloody fluid, approximately 2400 mL, was identified in the abdominal cavity. A blood clot was expelled from the omental foramen, and a larger clot was identified within the retroperitoneal omental sac. Bleeding was linked to the delicate structure of blood vessels resulting from tumor invasion and the rupture of the suture eye caused by increased arterial pressure in the celiac trunk. The tumor cells exhibited a spindle-shaped morphology characterized by abundant cytoplasm and abnormal nuclear division, as demonstrated by hematoxylin-eosin staining (Fig. 2A and B). Immunohistochemical staining demonstrated that the tumor cells exhibited partial cytoplasmic positivity for S-100 protein (Fig. 2C and D), focal cytoplasmic positivity for cytokeratin (CK) (Fig. 2E and F), and desmin (Fig. 2G and H). The findings for chromogranin A (CgA) and synaptophysin (Syn) were negative. The Ki-67 positivity score ranged from 40% to 60%.

Tissues acquired from unstained paraffin sections and cardiac blood collected during autopsy were subjected to genetic testing for precise tumor diagnosis.The patient displayed a missense mutation in *NF1*, identified by a nucleotide point mutation at position 1885 (G to A), leading to an amino acid substitution from glycine to arginine at position 629.

## 3. Discussion

The clinical manifestations of NF1 can be categorized into nonneoplastic or neoplastic types. Non-tumor symptoms of NF1 prominently include pigmentary lesions, specifically café-au-lait spots and Lisch nodules, alongside skeletal abnormalities and vascular disorders. The primary manifestation of tumors is primarily as neurofibromas, with a subset of individuals progressing to MPNST.^[9]^ In this case, the decedent exhibited numerous café-au-lait spots and neurofibromas on the skin, in addition to a retroperitoneal MPNST. Additionally, a genetic mutation in the *NF1* gene (1885G > A) and an amino acid variation (glycine 629 arginine) were identified in the heart blood and tumor tissue of the deceased. The decedent fulfilled the diagnostic criteria for NF1, as specified by the World Health Organization.

Histopathologically, MPNST are predominantly composed of densely arranged spindle-shaped tumor cells. The nuclei of tumor cells exhibit pronounced staining, and abnormal nuclear division is frequently noted. In 50% to 90% of patients, tumor cells demonstrate either scattered or localized positivity for S-100 protein.^[10]^ Additionally, diverse forms of heterogeneous differentiation, such as epithelial, myogenic, and neuroendocrine, are observed in 10% to 20% of cases.^[11]^ Epithelial markers, including CK, yield positive results in instances of epithelioid differentiation. Myogenic differentiation led to the identification of myogenic markers, including desmin. Chromogranin A and synaptophysin exhibit positive expression during neuroendocrine differentiation. The Ki-67 labeling index is instrumental in evaluating nerve sheath tumors in NF1 patients by identifying areas of significant cell proliferation. Ordinary and atypical neurofibromas generally demonstrate low labeling indices, between 2% and 5%. A proliferation rate exceeding 10% suggests the potential development of MPNST within a neurofibroma.^[12]^ In this case, the tumor cells demonstrated partial cytoplasmic positivity for S-100 protein and focal cytoplasmic positivity for CK and desmin. The Ki-67 positivity rate in the tumor cells varied between 40% and 60%. The findings substantiate the pathological diagnosis of MPNST exhibiting both epithelial and myogenic differentiations. In our opinion, it is essential to evaluate the potential for malignant transformation and heterologous differentiation comprehensively. This is particularly pertinent for deceased individuals who do not possess relevant medical documentation during their lifetime, to ensure they are not overlooked.

In patients with a history of NF1 or presenting typical symptoms, the presence of a rapidly growing tumor accompanied by neurological symptoms and/or discomfort raises suspicion for an MPNST.^[13]^ After the diagnosis of MPNST, surgical removal of the tumor remains the sole curative treatment option. For surgical success, complete excision of the tumor with a minimum of 2 cm negative margins is essential.^[14]^ Patients with insufficient tumor excision exhibit a significantly elevated risk of both local and distant recurrences compared to those who have received complete tumor removal.^[15]^ Complete tumor removal may be challenging in certain cases due to its position and size, complicating the prevention of postoperative complications.^[16]^ Adjuvant radiotherapy is advised for high-grade tumors exceeding 5 cm when total resection is unfeasible. In addition, due to neurofibromatous invasion of the tunica media, the fragility of blood vessels in patients with MPNST should be taken into account when selecting treatment, particularly during surgical or other invasive procedures near the great vessels. Consequently, meticulous postoperative monitoring is essential.^[17,18]^

In this case, forensic pathologists identified the patient as having a heterogeneous differentiated MPNST through the use of immunohistochemical staining and genetic testing. Regrettably, the clinical pathologists were unable to establish a definitive diagnosis. Clinical surgeons were indiscriminately opt for surgical intervention, which resulted in patient mortality due to hemorrhage post-surgery. Therefore, surgery may not be practical in instances where the tumor is sizable and situated in a difficult location. A comprehensive understanding of MPNST initiation and progression is essential for the advancement of novel treatments.

## Author contributions

**Investigation:** Yu Yang Pei, Tian Tong Yang.

**Methodology:** Hai Dong Zhang, Tian Shui Yu.

**Supervision:** Hai Dong Zhang.

**Validation:** Tian Tong Yang.

**Writing – original draft:** Yu Yang Pei.

**Writing – review & editing:** Tian Shui Yu.

## References

[1] Kallionpää RA Uusitalo E Leppävirta J Pöyhönen M Peltonen S Peltonen J. Prevalence of neurofibromatosis type 1 in the Finnish population. Genet Med. 2018;20:1082–6. 29215653 10.1038/gim.2017.215

[2] Miller DT Freedenberg D Schorry E Ullrich NJ Viskochil D Korf BR; COUNCIL ON GENETICS. Health supervision for children with neurofibromatosis type 1. Pediatrics. 2019;143:e20190660. 31010905 10.1542/peds.2019-0660

[3] Stalnecker CA Der CJ. RAS, wanted dead or alive: advances in targeting RAS mutant cancers. Sci Signal. 2020;13:eaay6013. 32209699 10.1126/scisignal.aay6013 PMC7393681

[4] Kolberg M Høland M Agesen TH. Survival meta-analyses for >1800 malignant peripheral nerve sheath tumour patients with and without neurofibromatosis type 1. Neuro Oncol. 2013;15:135–47. 23161774 10.1093/neuonc/nos287 PMC3548581

[5] Miao R Wang H Jacobson A. Radiation-induced and neurofibromatosis-associated malignant peripheral nerve sheath tumours (MPNST) have worse outcomes than sporadic MPNST. Radiother Oncol. 2019;137:61–70. 31078939 10.1016/j.radonc.2019.03.015

[6] Kim A Stewart DR Reilly KM Viskochil D Miettinen MM Widemann BC. Malignant peripheral nerve sheath tumours state of the science: leveraging clinical and biological insights into effective therapies. Sarcoma. 2017;2017:7429697. 28592921 10.1155/2017/7429697 PMC5448069

[7] Farid M Demicco EG Garcia R. Malignant peripheral nerve sheath tumours. Oncologist. 2014;19:193–201. 24470531 10.1634/theoncologist.2013-0328 PMC3926794

[8] Nakai S Uchida T Kuroda Y. Endovascular repair for abdominal aortic aneurysm rupture with neurofibromatosis type 1. Ann Vasc Surg. 2022;79:439.e1–4. 10.1016/j.avsg.2021.07.037 34648864

[9] Mitchell DK Burgess B White EE. Spatial gene-expression profiling unveils immuno-oncogenic programs of NF1-associated peripheral nerve sheath tumor progression. Clin Cancer Res. 2024;30:1038–53. 38127282 10.1158/1078-0432.CCR-23-2548 PMC11095977

[10] Patel S Pathak J Dekate K Mohanty N. Malignant peripheral nerve sheath tumour (MPNST) of mandible: solving the perplexity. BMJ Case Rep. 2015;2015:bcr2014207790. 10.1136/bcr-2014-207790 PMC4693118 25762575

[11] WHO Classification of Tumours Editorial Board. WHO classification of tumours of soft tissue and bone: WHO classification of tumours. 2013;5:187–89.

[12] Miettinen MM Antonescu CR Fletcher CDM. Histopathologic evaluation of atypical neurofibromatous tumors and their transformation into malignant peripheral nerve sheath tumor in patients with neurofibromatosis 1-a consensus overview. Hum Pathol. 2017;67:1–10. 28551330 10.1016/j.humpath.2017.05.010 PMC5628119

[13] Martin E Coert JH Flucke UE. Neurofibromatosis-associated malignant peripheral nerve sheath tumours in children have a worse prognosis: a nationwide cohort study. Pediatr Blood Cancer. 2020;67:e28138. 31889416 10.1002/pbc.28138

[14] Gupta G Mammis A Maniker A. Malignant peripheral nerve sheath tumours. Neurosurg Clin N Am. 2008;19:533–43, v. 19010279 10.1016/j.nec.2008.07.004

[15] Dunn GP Spiliopoulos K Plotkin SR. Role of resection of malignant peripheral nerve sheath tumours in patients with neurofibromatosis type 1. J Neurosurg. 2013;118:142–8. 23101443 10.3171/2012.9.JNS101610

[16] Levi AD Ross AL Cuartas E Qadir R Temple HT. The surgical management of symptomatic peripheral nerve sheath tumours. Neurosurgery. 2010;66:833–40. 20190660 10.1227/01.NEU.0000367636.91555.70

[17] Higa S Nagano T Ito J. Three arterial ruptures in a patient with neurofibromatosis type 1. Ann Vasc Dis. 2021;14:168–72. 34239644 10.3400/avd.cr.20-00174 PMC8241549

[18] Omura T Ikawa K Kudo E. Acute hemorrhagic cholecystitis related to diffuse neurofibroma of gallbladder in a patient with neurofibromatosis type 1. Surg Case Rep. 2023;9:62. 37079137 10.1186/s40792-023-01647-2 PMC10119345

